# COVID-19 and dermatology

**DOI:** 10.3906/sag-2005-182

**Published:** 2020-12-17

**Authors:** Ülker GÜL

**Affiliations:** 1 Department of Dermatology, Gülhane Faculty of Medicine, Health Sciences University, İstanbul Turkey

**Keywords:** SARS-CoV-2, COVID-19, dermatology, skin findings

## Abstract

**Background/aim:**

Sars-CoV-2 virus infection (COVID-19) was observed in China in the last months of 2019. In the period following, this infection spread all over the world. In March 2020 the World Health Organization announced the existence of a pandemic.

The aim of this manuscript is to investigate skin diseases associated with COVID-19 under three main headings: skin problems related to personal protective equipment and personal hygiene measures, skin findings observed in SARS-CoV-2 virus infections, and skin findings due to COVID-19 treatment agents.

**Materials and methods:**

In PubMed, Google Scholar databases, skin lesions related to personal protective equipment and personal hygiene measures, skin findings observed in SARS-CoV-2 virus infections and skin findings due to COVID-19 treatment agents subjects are searched in detail.

**Results:**

Pressure injury, contact dermatitis, itching, pressure urticaria, exacerbation of preexisting skin diseases, and new skin lesion occurrence/new skin disease occurrence may be due to personal protective equipment. Skin problems related to personal hygiene measures could include itching, dryness, and contact dermatitis. Skin findings may also be observed in SARS-CoV-2 virus infections. The incidence of skin lesions due to COVID-19 was reported to be between 0.2% and 29%. Many skin lesions including maculopapular, urticarial, vesicular, chilblain-like, thrombotic/ischemic, etc. are observed in COVID-19 patients. Some authors have stated that there is an absence of SARS-CoV-2 virus infection-specific skin findings. However, in asymptomatic or presymptomatic COVID-19 patients in particular, skin lesions can lead to the diagnosis of COVID-19. In addition, skin lesions may occur due to COVID-19 treatment agents.

**Conclusion:**

Many skin lesions may appear as a result of COVID-19. Even in the absence of a COVID-19 diagnosis, skin findings should be evaluated carefully in this pandemic period.

## 1. Introduction

SARS-CoV-2 virus infection (COVID-19) was observed in China in the last months of 2019. In the period following, this infection spread all over the world. In March 2020 the World Health Organization announced the existence of a pandemic [1–3].

Skin is the largest organ in our body. Factors such as external changes, infectious agents, internal events, and medications can cause symptoms in the skin. This manuscript presents a review of the literature for COVID-19 and dermatology.

## 2. Occupational exposure of dermatologists to SARS-CoV-2 virus

Some cases of occupational exposure are symptomatic or even serious, and some cases exhibit mild symptoms or none at all. Therefore, especially in mild or asymptomatic cases, skin findings may lead to the diagnosis of COVID-19 [1–7].

PCR false-negative cases were found in 33%–41% of COVID-19 cases [6,7]. Furthermore, skin lesions may also precede COVID-19 symptoms [8].

Dermatological examinations require the examination of body areas such as the face, oral mucosa, etc. while being in close contact with the patient [9,10]. For this reason, COVID-19 diagnosed patients may be a source of infection during the pandemic period. In addition, due to the presence of asymptomatic cases or COVID-19 carriers, dermatologists may also be infected. Dermatologists should be careful when preparing for examinations and should always wear personal protective equipment and obey personal hygiene rules [10–14].

## 3. COVID-19 and skin diseases

In this manuscript, skin diseases associated with COVID-19 are investigated under the following headings:

**3.1. Skin lesions related to personal protective equipment (PPE) and personal hygiene (PH) measures,**

**3.2. Skin findings observed in SARS-CoV-2 virus infections,**

**3.3. Skin findings due to COVID-19 treatment agents.**

### 3.1. Skin lesions related to personal protective equipment and personal hygiene measures

During the pandemic period, all people have been practicing heightened personal hygiene (frequent disinfectant use and washing). Healthcare workers in particular have been exposed to the regular use of PPE such as the N95 mask, gloves, goggles, and gowns [13–20]. Skin problems related to PH measures and PPE were most frequently observed in the hands (15%–85%) and face (12%–87%). Less frequently, they were seen on the legs, trunk, and all over the body. As the frequency of PH application increases and PPE usage time increases (in hours and days), the incidence of skin lesions increases. Depending on the degree of protection and hygiene, among health care workers 11%–89% reported erythema, 13%–87% papule/edema, 9%–91% exudation/crust, 12%–88% scratches, 12%–88% fissure, 30%–70% lichenification, and 16%–84% blisters [15]. According to one questionnaire 49.0% of health workers reported mask-related skin reactions, and among these 41.8% had facial skin problems and 6.2% had eye symptoms [16].

Skin lesions observed due to prolonged contact with PPE and excessive PH are listed in Table 1 [15–30].

**Table 1:** Skin lesions related to personal protective equipment and personal hygiene measures.

Protectiontype	Personal protective equipment		Personal hygiene measures
Protectionagents	N95 masks (mask and stems), goggles and face shields	N95 and surgical masks, goggles,gowns, gloves	Disinfectants, soaps, frequent washing
Diseases	· Pressure injury· Pressure urticaria	· Contact dermatitis· Itching· Exacerbation of preexisting skin diseases· New skin disease	· Contact dermatitis· Itching· Exacerbation of preexisting skin diseases· New skin disease

#### 3.1.1. Pressure injury

The N95 masks, goggles, and face shields used as PPE can squeeze and rub the cheeks, forehead, and nasal bridge, which can easily cause mechanical damage to the skin. As a result, ecchymosis, maceration, abrasion, and erosion have been observed. The nasal bridge was the most commonly affected area (83.1%). In addition, lesions may occur where the stems of N95 masks create pressure, such as the ears [10,18–20,22–25]. In one study, facial indentation was observed in 18.8% of cases, and lesions caused by mask stems were observed in 22.3% of cases; 8.9% (n: 36) of those affected removed their masks because they could not tolerate them [16]. Skin indentation may be mild or serious. Most mild skin indentations regress spontaneously; however, if the ulceration is not properly managed, secondary infections may occur [10].

#### 3.1.2. Contact dermatitis (CD)

##### 3.1.2.1. Face

CD is one of the important problems to emerge due to mask use. Lesions often occur on the nose and cheeks [26]. Both N95 and surgical masks contain formaldehyde and other preservatives. For this reason, allergic contact dermatitis (ACD) may occur. Friction, warmth, and moisture from respiration may enhance these symptoms. Skin barrier dysfunction and skin microbiota disorder make patients more vulnerable to mask side effects [16,27,28].

##### 3.1.2.2. Body

Skin dermatoses most commonly develop where gowns adhere tightly to the skin. Friction, moisture, and warmth in these regions may increase the risk of ACD [25].

##### 3.1.2.3. Hands

CD has frequently been observed in the hands during the pandemic period. Hand hygiene is extremely important. The causes of CD in the hands are frequent contact with disinfectants and frequent washing. After exposure to 60%–80% alcohol, chlorine-based disinfectants, peroxyacetic acid, and chloroform users may develop adverse reactions (e.g., irritant CD). In addition, the frequency and duration of skin cleaning has increased. Excessive hygiene applications also increase CD [10,19,26]. Atopic diathesis, low humidity, frequency of hand washing, wet work, glove use, and duration of work are important risk factors for the development and/or aggravation of hand dermatitis. Hand dermatitis often appears in the form of irritant CD. Less commonly, ACD may occur. For the aim of CD prevention, applying hand cream frequently is recommended [18,21,30]. Excessive washing of the skin and repeated application of disinfectants (e.g., bleach and alcohol) should be avoided [19].

Prolonged use of gloves also causes CD. In one study, dry skin (73.4%), itching (56.3%), and rash (37.5%) were reported due to the use of permanent gloves [17].

#### 3.1.3. Itching

Itching is observed at rates between 14.8% and 55.2% depending on the degree of protection. The incidence of itching was 79% when protection was in use for more than 3 days. Itching was observed at the rate of 37% when using PPE 0–4 h a day and 67% when using PPE for more than 4 h. This is especially relevant for healthcare professionals [15]. In one study, mask-related itching was observed at a rate of 15% [16]. In another study, facial itching was reported at 51% due to mask use, and itching of the hands was reported at 56.3%, due to glove use [17].

#### 3.1.4. Pressure urticaria

Pressure urticaria is rarely observed [10,27,29]. Antihistamines such as cetirizine and loratadine are preferred for treating pressure urticaria, and antileukotriene agents may be added if needed [10].

#### 3.1.5. Exacerbation of preexisting skin diseases including seborrheic dermatitis and acne

In one study, exacerbation was reported in 43.6% (n = 44) of acne patients, in 37.5% (n = 9) with seborrheic dermatitis, and in all 14 acne rosacea patients [16]. Prolonged wearing of masks and goggles may aggravate existing acne vulgaris. Plausible mechanisms include the rupture of comedones induced by pressure and friction, occlusion of the pilosebaceous duct, microcirculation dysfunction due to long-term pressure, and a humid environment which is conducive to bacteria proliferation [10].

#### 3.1.6. New skin lesion occurrence/new skin disease occurrence

Frequent disinfection of the hands and the wearing of latex gloves may result in pompholyx presenting with blisters and itching. The long-term wearing of protective clothing may cause sweating, which can lead to dermatitis and folliculitis. Frequent use of shoe covers may cause fungal infections of the feet (19). The regular use of N95 masks has been found to cause acne (59.6%) [17,25].

### 3.2. Skin findings observed in SARS-CoV-2 virus infections

The reported incidence of skin lesions was 0.2% in 1099 cases of SARS-CoV-2 infection in China [31,32]. In another study, this rate was 4.9% (5/103) [33]. Skin rash was reported in 29% (14/48) of patients in an Italian study [34]. In another Italian study, cutaneous manifestations were reported in 20.4% (18/88) of confirmed COVID-19 patients [35].

Many skin lesions are observed in COVID-19 (Table 2) [8,36,37]. The largest series (373 cases) observing skin lesions was from Galván Casas et al. [8]. Some authors stated that there were no SARS-CoV-2 virus infection-specific skin findings [38]. However, some skin lesions may help to diagnose COVID-19 [8,38].

Time to onset of skin lesions: The relationship between skin lesions and the primary symptoms of COVID-19 has been studied and in one study 5.9% of cases show skin lesions before primary symptoms, 56.8% cases show lesions with the primary findings, and 37.3% show lesions after the primary symptoms (Table 3) [8]. In a study analyzing18 publications, in 12.5% (9/72) of the patients, skin lesions were observed before the onset of respiratory symptoms or COVID-19 diagnosis [39]. As a result, particularly in asymptomatic or presymptomatic COVID-19 patients, skin lesions can lead to a diagnosis of COVID-19 [8].

The relationship between skin lesions and disease severity: There is no evidence that the extent of cutaneous involvement is related to disease severity [38,39]. On the other hand, in 2 separate reports COVID-19 and skin lesion severity were linked in 2 patients [34,36].

Age and gender characteristics of skin lesions: In a study reporting on 18 articles and 3 additional cases, mean patient age was 53.6 years. In this study, males accounted for 38.9% of cases, females accounted for 27.8% of cases, and gender was not reported in 37.5% of cases [39].

Location of lesions: In an evaluation of publications, the majority of lesions were localized on the trunk (66.7%, 50/72); however, 19.4% (14/72) of patients experienced cutaneous manifestations on the hands and feet [39].

Skin lesion healing time: Generally, lesions spontaneously healed in all patients within 10 days. The majority of studies reported no correlation between COVID-19 severity and skin lesions [39].

Histopathologic examination: In most of the literature, histological findings were not reported [39].

**Skin findings observed in COVID-19**

#### 3.2.1. Maculopapular exanthema (MPE)

The incidence of MPE is seen in Table 2, and time of occurrence is in Table 3 [8,33,35]. In a study analyzing 18 publications, MPE was the most common cutaneous manifestation of COVID-19 (36.1%) [39]. Maculopapular exanthema may also be observed during the asymptomatic period [8]. It has also been seen in children [40].

**Table 2:** Incidence of skin lesions in all COVID-19 cases and among all COVID-19 skin findings.

Study	Maculapapular (%)		Urticarial (%)		Vesicular (%)	
	All COVID-19 cases	All skinfindings	All COVID-19 cases	All skinfindings	All COVID-19 cases	All skinfindings
Recalcati [35]	15.9	77.8	3.4	16.7	1.1	5.6
Hedou [33]	1.9	40	1.9	40		
Galván Casas [8]		47		19		9
Zhang [45]			1.4			
Tammaro [49]					1.5	

**Table 3:** The relationship of skin lesions with the main symptoms of COVID-19 [8].

Lesion development time	Pseudo-chilblain	Vesicular	Urticarial	Maculopapular	Livedo/ necrosis	Total
Before n (%)	5 (7)	5(15)	3(4)	8(5)	1(5)	22 (5.9)
Synchronously n (%)	24(34)	19(56)	43(61)	108(61)	18(86)	212 (56.8)
After n (%)	42(59)	10(29)	25(35)	60(34)	2(10)	139 (37.3)
Total	71(19)	34(9.1)	71(19)	176(47.2)	21(5.6)	373

Maculopapular exanthema accompanying COVID-19 is nonspecific (some cases have a purpuric component). Scaling may be found, and itching is present in half of the patients [8,37,41]. Purpura may also be present, either punctiform or over larger areas [8,39,41,42]. Lesions may be located in perifollicular areas. Target-like purpuric or pseudovesicular plaques can also be seen [8,43,44]. Some were described as similar to pityriasis rosea, erythema elevatum diutinum, or erythema multiforme. Differential diagnosis should be performed to account for other infections and drug reactions [8].

#### 3.2.2. Urticarial lesions

Urticarial lesions incidence is listed in Table 2, and time of occurrence is in Table 3 [8,33,35,45]. In an analysis of 18 articles, urticaria was found in 9.7% of cases [39]. Urticaria may be the first sign of COVID-19 [8,46,47]. Urticaria was also seen during the asymptomatic phase [8,33]. It has also been reported in children [40]. Urticaria is mainly located on the face and upper body [33]. It is mostly distributed on the trunk or disperse. A few cases were palmar. Itching was very common when urticariform lesions were present (92%) [8]. A case of cold urticaria has also been reported in the literature [42]. Differential diagnosis should be performed to rule out other infections and drug reactions [8].

Zhang et al. found urticaria in 1.4% of COVID-19 patients. In this study, there were 82 nonsevere patients, 58 severe patients, and a total of 140 cases. Urticaria was observed in 1.2% of nonsevere patients and in 1.7% of severe patients [45].

In the literature, the histologic examination of a female case with a urticarial lesion revealed a perivascular infiltrate of lymphocytes, some eosinophils, and upper dermal edema. Oral antihistamines were added to her treatment and she improved over a 5-day period [48].

#### 3.2.3. Vesicular eruptions

The incidence of vesicular eruptions is seen in Table 2, and the time of occurrence is in Table 3 [8,35,49]. Vesicular eruptions have also been observed during the asymptomatic phase [8]. In one study analyzing 18 publications, papulovesicular rash was found in 34.7% of cases [39].

Some lesions were on the trunk and consisted of small monomorphic vesicles that were chickenpox-like. Lesions appeared in middle aged patients. Itching was common (68%) [8]. Two out of 130 patients had isolated herpetiform lesions appear on their trunks during their inpatient stay [49]. In the lesions, vesicles surrounded by erythematous halos were observed [49].

#### 3.2.4. Acral lesions

Acral lesions in COVID-19 are mentioned in different publications under different names [8,37,50–56]. Galván Casas et al. have divided acral lesions into 2 groups as follows [8]:

##### 3.2.4.a. Chilblains-like lesions (perniosis-like lesions and pseudo-chilblain)

The cutaneous manifestations consisted of erythemato-violaceous papules and macules, with possible vesicles, bullous, or pustules on the acral area [8,32,53,54]. These lesions resembled chilblains and had purpuric areas, affecting the hands and feet [8,53]. They were usually asymmetrical [8]. In an analysis of 18 publications, chilblains-like lesions were found in 19% of cases [39]. They occurred before the main symptoms in 7% of the cases. (Table 3) [8]. In another study, lesions were found in 25.4% of asymptomatic patients [55]. Chilblains-like lesions appeared more commonly after the onset of the disease and were not associated with severe disease [8]. The appearance time of the lesions was 10 days after the onset of the disease [55]. They may cause pain (32%) or itching (30%). Chilblains-like lesions affected children and teenage patients at a higher rate [8,51,53,55]. Chilblains in youth are a potential sign of COVID-19 infection [52]. No significant difference in gender was noticed [55]. No association with cold exposure, comorbidities, or drug intake was recorded [53]. In 63 cases, a history of autoimmune disorders was found in only 6 patients (one with ANA positivity), while familial or personal history of coagulation defects was seen in 4 cases [55]. In another study, antinuclear antibody, C3 and C4, anticardiolipin antibodies, and cryoglobulin were negative [54]. In contrast, Andina et al. found that coagulation tests and lupus anticoagulant levels were normal in all patients tested. These lesions were observed in mildly symptomatic COVID-19 patients. In addition, chilblains-like lesions had an excellent prognosis, usually requiring no therapy [51]. In another publication, the appearance of chilblains associated with Raynaud’s phenomenon was reported in one case [42]. Histology of the lesions showed a mild superficial +/– deep perivascular dermatitis [53]. The following histological findings were found in a 23-year-old male COVID-19 patient with chilblains lesions; no intraluminal fibrin thrombi were identified, no fibrin was identified within venule walls, and direct immunofluorescence results were negative [56].

##### 3.2.4.b. Acral ischemia (livedo or necrosis) (6%)

When evaluated according to the onset of the main symptoms, acral ischemia developed prior to onset in 5% of the cases, synchronously in 86% of the cases, and later in 10% of cases (Table 3). These patients showed different degrees of lesions suggesting occlusive vascular disease including areas of acral ischemia. Livedoid/necrotic lesions appeared in older patients with more severe disease (10% mortality) [8]. In one study analyzing 18 publications, painful purple papules in the acral area were found in 19% of cases, and livedo reticularis was found in 2.8% of cases [39]. In one study, 7 patients were reported to have acro-ischemia findings including finger/toe cyanosis, skin bulla, and dry gangrene. D-dimer, fibrinogen, and fibrinogen degradation product (FDP) were significantly elevated in most patients. Prothrombin time was prolonged in 4 patients. D-dimer and FDP levels increased progressively when exacerbated by COVID-2019, and 4 patients were diagnosed with definite disseminated intravascular coagulation [57]. Thrombosis-related livedoid lesion and bulla may develop in patients [58]. Zhang et al. reported three cases of thrombosis associated with antiphospholipid antibodies [59]. Another study found abnormal coagulation parameters were associated with poor prognosis in patients [60].

#### 3.2.5. Other lesions observed in COVID-19

· Erythema multiforme-like lesions (EMLL): EMLL was observed in 4 female patients hospitalized due to COVID-19; the mean age of patients was 66.75 years (range 58–77). The mean time period between the onset of COVID-19 symptoms and the appearance of cutaneous lesions was 19.5 days (range 16–24). Typical target lesions were observed in two patients. Lesions were on the back and then spread to the face and limbs within 1 week, without involvement of palms or soles. All patients were treated with systemic corticosteroids and had progressive resolution of the skin lesions within 2–3 weeks. Histopathological examination showed nonspecific findings. Three patients had their oral cavity examined, which revealed palatal macules and petechiae [61]. It was reported that a female COVID-19 patient had generalized exanthematous pustulosis with erythema multiforme-like lesions [62]. In another paper it was found that atypical erythema multiforme palmar plaques lesions were formed due to Sars-Cov-2 [63].

· Vasculitis [64]

· Transient livedo reticularis [65]

· Nonpruritic annular fixed plaques: In a 39-year-old male with a fever of 39 °C, nonpruritic annular fixed plaques were observed. This rash was located on the upper limbs, chest, neck, abdomen, and palms, sparing the face and mucous membranes. Blood count, electrolytes, C-reactive protein, and anti-DNA antibodies were at normal levels. Histological findings were unspecific but consistent with viral exanthemata [66].

· Acute hemorrhagic edema [43,44]

· Dengue fever-like [40,44]

· Venous thromboembolism: The incidence of venous thromboembolism was 25% (20/81) in severe novel coronavirus pneumonia patients [67].

· An increased number of herpes zoster and superficial fungal infection cases were observed in COVID-19 patients [8].

· Oral herpes simplex virus type 1 reactivation was seen in an intubated patient in intensive care [33].

#### 3.2.6. Oral mucosa lesions

In three cases (two suspected and one confirmed), painful ulcers and blisters in the oral cavity (desquamative gingivitis) were observed [68]. Galván Casas et al. also reported enanthem in COVID-19 cases [8,68].

### 3.3. Skin findings due to COVID-19 treatment agents

The treatment agents for COVID-19 can also cause skin lesions. In cases of COVID-19 with skin lesions, drug eruptions should be considered in the differential diagnosis. Below are the cutaneous side effects of the treatment agents used in COVID-19.

#### 3.3.1. Hydroxychloroquine (HCQ)

Hydroxychloroquine is an antimalarial agent which also has antiinflammatory and immunomodulatory activities. Hydroxychloroquine is used for short term COVID-19 treatment. However, some people use HCQ for long-term treatment without a doctor’s recommendation due to COVID-19 phobia. For these reasons, all patients having skin lesions should be questioned about their use of HCQ during the pandemic period. HCQ may cause adverse cutaneous drug eruptions (ACDRs). In 6 of 180 patients diagnosed with cutaneous lupus erythematosus and dermatomyositis ACDRs have been observed. ACDRs occurred 5 to 14 days after initiation of HCQ treatment and none were life threatening. Eruptions were characterized as lichenoid, urticarial, or exanthematous and resolved after discontinuation of HCQ treatment [69]. In one case, generalized pustular figurate erythema was seen [70]. One of the important side effects of HCQ is hydroxychloroquine-induced pruritus [71]. Moreover, it may cause acute generalized exanthematous pustulosis, urticaria, mucocutaneous dyspigmentation, Stevens–Johnson‐like eruptions, alopecia, the bleaching of hair, and psoriasis flare-ups [72].

#### 3.3.2. Azithromycin

Azithromycin is widely used and is generally considered a safe medicine. Azithromycin rarely causes skin reactions such as Bullous fixed drug eruption, Stevens–Johnson syndrome, DRESS, leukocytoclastic vasculitis, hypersensitivity syndrome, etc. [73–77].

#### 3.3.3. Favipiravir

Favipiravir is a nucleoside analog that is well-known as a broad-spectrum antiviral drug. In the commercial drug prospectus, skin rash (< 1%) and eczema (<0.5%) pruritus are reported. In COVID-19 treatment with favipiravir, cutaneous adverse effects have not been observed [78].

#### 3.3.4. Remdesevir

Remdesivir is a monophosphoramidate prodrug of an adenosine analog. Some adverse skin effects have been reported including itching and swelling (especially of the face, tongue, and throat). In one study, Wang et al. found skin rash in 7% of the remdesevir group and 3% of a placebo group of COVID-19 patients. Remdesevir was discontinued due to cutaneous adverse effects in 2 cases (1%) [79]. In another study, skin rash was observed in a total of 4 cases [3 cases (9%) in invasive ventilation and 1 case (5%) in noninvasive oxygen support] [80].

#### 3.3.5. Oseltamivir

Oseltamivir has been approved for influenza treatments and may cause cutaneous side effects such as angioedema, Stevens–Johnson syndrome, toxic epidermal necrolysis, etc., however this is rare [81–85].

#### 3.3.6. Combination of lopinavir/ritonavir

Lopinavir/ritonavir is an HIV-1 protease inhibitor. Cutaneous adverse effects are given below [86–88].

· Common: maculopapular rash, eczema, seborrheic dermatitis, night sweats, and pruritus,

· Uncommon: alopecia, capillaritis, and vasculitis,

· Rare: Steven–Johnson syndrome, erythema multiforme, and acute generalized exanthematous pustulosis.

## 4. Conclusion

Many skin lesions may appear during the COVID-19 pandemic period: skin lesions related to personal protective equipment and personal hygiene measures, skin findings observed in SARS-CoV-2 virus infections, and cutaneous drug eruption due to COVID-19 treatment agents. Even in the absence of a COVID-19 diagnosis, skin findings should be evaluated carefully in this pandemic period. In asymptomatic or presymptomatic COVID-19 patients, skin lesions can lead to the diagnosis of COVID-19.
